# Development of a multiplex and sensitive lateral flow immunoassay for the diagnosis of periprosthetic joint infection

**DOI:** 10.1038/s41598-019-52051-6

**Published:** 2019-10-30

**Authors:** Tsung-Ting Tsai, Tse-Hao Huang, Natalie Yi-Ju Ho, Yu-Pei Chen, Chung-An Chen, Chien-Fu Chen

**Affiliations:** 1 Department of Orthopaedic Surgery, Bone and Joint Research Center, Chang Gung Memorial Hospital and Chang Gung University College of Medicine, Taoyuan, 333 Taiwan; 2 Institute of Applied Mechanics, National Taiwan University, Taipei, 106 Taiwan

**Keywords:** Medical and clinical diagnostics, Biomedical engineering

## Abstract

The diagnosis of periprosthetic joint infection (PJI) remains a challenge. However, recent studies showed that synovial fluid biomarkers have demonstrated greater diagnostic accuracy than the currently used PJI diagnostic tests. In many diagnostic tests, combining several biomarkers into panels is critical for improving diagnostic efficiency, enhancing the diagnostic precision for specific diseases, and reducing cost. In this study, we prove that combining alpha-defensin and C-reactive protein (CRP) as biomarkers possesses the potential to provide accurate PJI diagnosis. To further verify the result, we developed a multi-target lateral flow immunoassay strip (msLFIA) with staking pad design to obtain on-site rapid response for clinical diagnosis of PJI. A total of 10 synovial fluid samples were tested using the msLFIA, and the results showed that the combined measurements of synovial fluid alpha-defensin and CRP levels were consistent with those obtained from a commercial enzyme-linked immunosorbent assay kit. In addition, we developed a multi-target lateral flow immunoassay strip (msLFIA) with staking pad design to obtain on-site rapid response for clinical diagnosis of PJI, which the multi-target design is used to increase specificity and the stacking pad design is to enhance detection sensitivity. As a result, the turnaround time of the highly sensitive test can be limited from several hours to 20 min. We expect that the developed msLFIA possesses the potential for routine monitoring of PJI as a convenient, low-cost, rapid and easy to use detection device for PJI.

## Introduction

Prosthetic-joint replacement is used to improve patients’ quality of life which includes providing symptom relief, increasing mobility, and restoring joint function. Thus, prosthetic-joint replacement is a safe and cost-effective surgical technique. Nonetheless, prosthetic joint infection (PJI) remains one of the most common indications for revision hip and knee arthroplasties^1–3^. Despite the wide variety of tests available for diagnosing PJI, the diagnosis of PJI still poses considerable clinical challenge^4,5^. In this study, joint aspiration in combination with clinical, radiological, and biochemical findings^6^, as well as serum C-reactive protein (CRP) and erythrocyte sedimentation rate were investigated^7^. In the investigation, synovial white blood cell count^8^, intraoperative findings included frozen section, presence of purulence, and isolation of a pathogen by culture^9,10^, are the standard tests for PJI diagnosis. Nevertheless, these available tests for PJI diagnosis also have disadvantages, including the cost, the need for specialized equipment, and the relatively long time taken to complete the analysis. Furthermore, none of these diagnostic tests have satisfactory efficacy, sensitivity, or specificity^11,12^.

In recent years, synovial fluid biomarkers, which includes leukocyte esterase^13,14^, alpha-defensin^15,16^, and CRP^17,18^, have been intensively studied for their potential in developing a rapid and accurate diagnosis of PJI. Regardless of the condition, individual biomarkers have been shown to lack the specificity and sensitivity necessary for their use as diagnostic tools in clinical settings. For example, the diagnostic sensitivity and specificity of alpha-defensin for PJI were from 64% to 100% and 82% to 100%, respectively^15,19–28^. On the other hand, the diagnostic sensitivity for CRP was from 70% to 85% and its specificity for PJI was from 85% to 95%^17,18,29,30^. Despite this, most PJI biomarker researches have been focusing on a single biomarker’s association with PJI instead of several biomarkers’ joint relationship with PJI. Combining different markers into a panel has been suggested to enhance PJI diagnostic performance^21,31^. Panels have previously been displayed to increase diagnostic performance significantly in several different diseases such as acute myocardial infarction, lung cancer, and prostate cancer^32–34^. Furthermore, it has been suggested that combinations of different biomarker types, for instance, inflammation proteins of CRP and antimicrobial peptide of alpha-defensin, can improve classification^35,36^. Thus, multiplexing is a critical parameter for increasing diagnostic efficiency, improving the diagnostic accuracy for specific diseases, and reducing diagnostic cost. Compared to standard laboratory technologies such as enzyme-linked immunosorbent assay (ELISA) and real-time polymerase chain reaction, which allow high-throughput and low volume processing but require expensive equipment, skilled personnel, and long processing time, lateral flow immunoassay (LFIA) based on chromatography and gold nanoparticles is the most widely used point of care testing (POCT) technology owing to its simple operation, rapid detection, and robustness in various applications. Therefore, LFIA becomes the focus of current health monitoring device research^37^. However, routine LFIA can only detect one target molecule at one time, whereas the multiplexing format of LFIA can simultaneously detect several target markers in a single strip. Moreover, running multiple assays may have subtle variations in each run, which can affect the results. On the other hand, running one single assay can avoid the variations from multiple assays so that any difference observed in the expression levels of multiple proteins is more likely to reflect the true abundances in the sample. Other advantages include less required sample volumes which can reduce the pain and the risk of infection from specimen collection, and rapid determination of multiplex test results with the naked eye. In addition, the costs of LFIA production include the materials and chemicals used in each strip to prevent matrix interference and to guarantee long-term stability, and also the fabrication of strips that involves manual work is very time-consuming. Therefore, a multiplex LFIA strip not only can reduce the operating cost but also improve the detection efficiency^38^.

In this study, we introduced a multi-target lateral flow immunoassay strip (msLFIA), which added an additional membrane between the conjugation pad and the test pad to the conventional AuNP-based LFIA format to enhance the detection sensitivity^39^, for measuring the protein levels of alpha-defensin and CRP in synovial fluid samples from patients undergoing revision arthroplasty for septic or aseptic failure. In the previous study^39^, synovial fluid sample was pre-treated with a 1:100 dilution in PBS buffer because the pure synovial fluid is viscous and difficult to flow through the strip completely; however, in this study, we performed a quick preincubation of mixing the pure synovial fluid sample with the AuNPs-labeled antibodies, so the protein levels in synovial fluid are specific for analyte without dilution. We aimed to develop a multiplex LFIA for rapid and high performance for PJI diagnosis.

## Results

**Figure 1:**
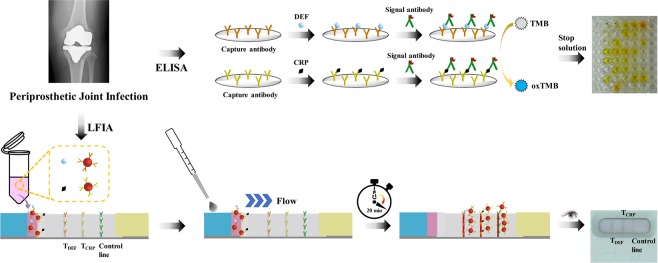
The schematic illustration of the proposed multi-target lateral flow immunoassay strip for rapid PJI diagnosis in the point of need.

In this study, we chose to combine biomarker measurements of alpha-defensin and CRP for more accurate PJI diagnosis. To further verify the result, we developed a multi-target lateral flow immunoassay strip with highly sensitive staking pad design to obtain on-site rapid response for clinical validation in the diagnosis of PJI. The ELISA tests were also performed to confirm the reliability of the msLFIA (Fig. 1).

### Optimization of the LFIA strips

**Figure 2:**
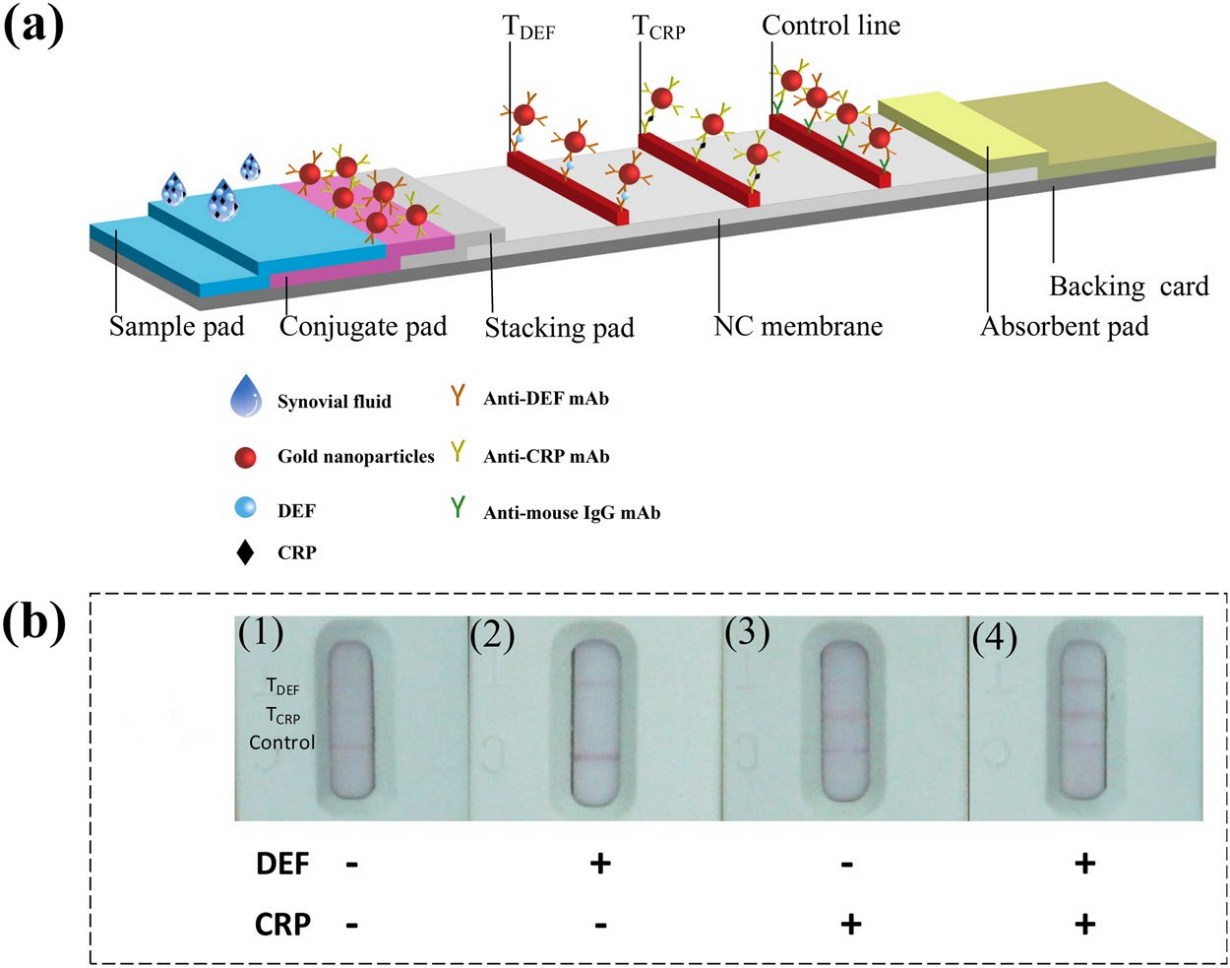
(**a**) Scheme of the msLFIA for alpha-defensin and CRP detection. (**b**) Qualitative analysis platform for the msLFIA. Strips 1 to 4 are the schematic illustrations of detection results. (1) DEF (−), CRP (−); (2) DEF (+), CRP (−); (3) DEF (−), CRP (+); (4) DEF (+), CRP (+).

In our msLFIA, the presence of the corresponding visible test line in the presence of a visible control line was used to define a sample as LFIA positive (Fig. 2). The presence of a visible control line and the absence of a visible test line was used to define a sample as LFIA negative (Fig. 2(b)). Each test line has a cutoff, which is the minimum concentration of target biomarker in the sample that is necessary for the test line to become visible, and this cutoff can be adjusted by varying the density of the capture antibody applied. To increase the sensitivity, accuracy, and reproducibility and avoid the difficulty of stabilizing dried reagents of the LFIA strip, a preincubation method was used^40^. Briefly, an equal volume (5 μL) of two different conjugates of AuNP-anti-alpha-defensin and -CRP were pre-mixed together. Afterwards, 1 μL of synovial fluid sample and 10 μL of AuNP-conjugate mixture were incubated in a tube prior to testing on the conjugate pad and was soon followed by dropping running buffer onto the LFIA strip, instead of coating them on conjugate pads and dried afterward. The red colored lines were visualized within 20 min, which is the reaction time recommended for LFIA that has high signal stability and good assay sensitivity.

### Performance of the msLFIA with synovial fluid

**Figure 3:**
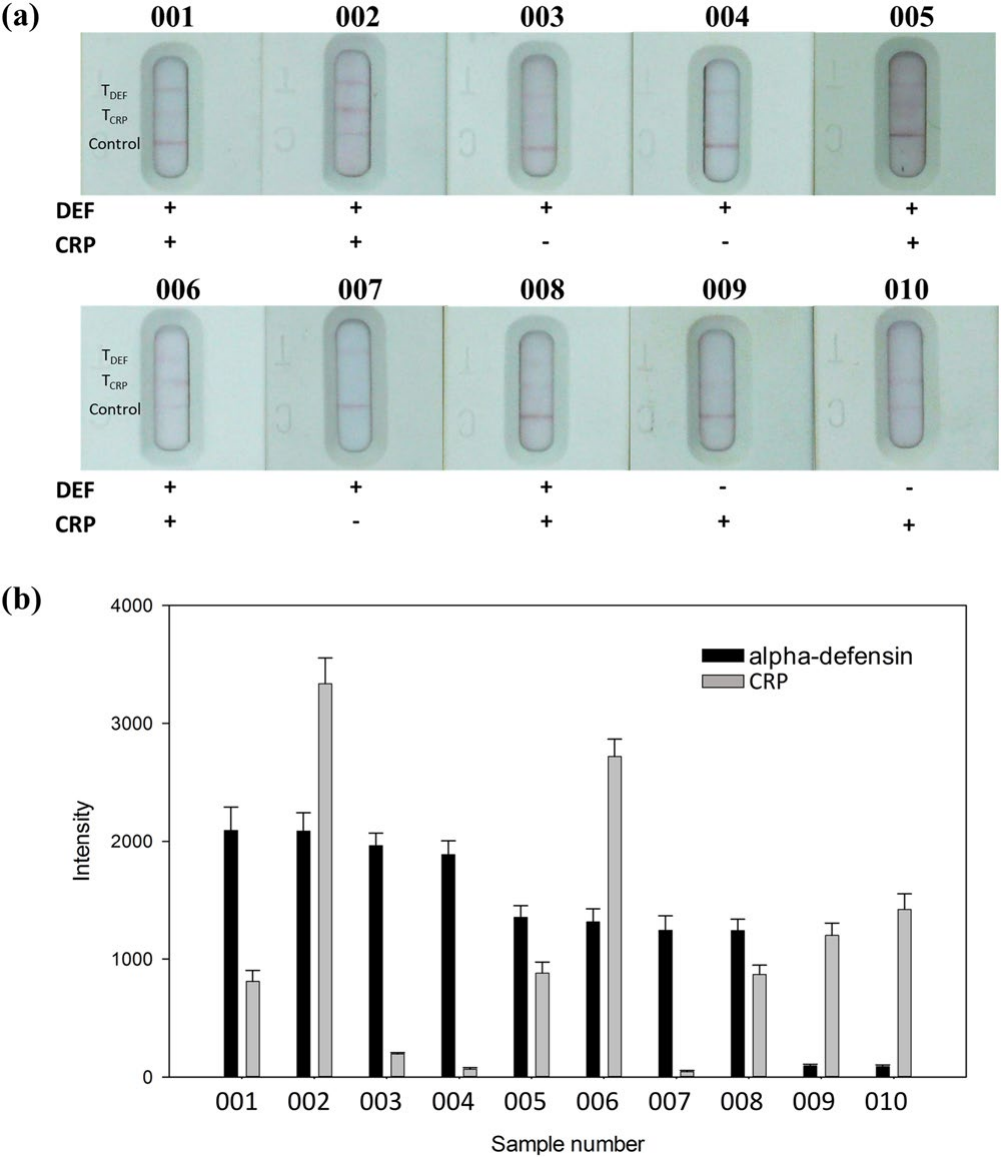
Alpha-defensin/CRP test results of the synovial fluid samples by msLFIA. (**a**) The captured image of the test results. (**b**) The optical intensity of synovial fluid samples analyzed directly using ImageJ. The error bars represent the standard deviation of three independent experiments.

**Figure 4:**
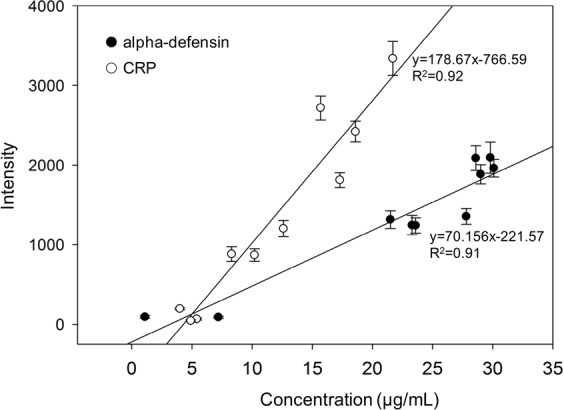
Correlation between the signal intensities of msLFIA results and ELISA measurements of CRP and alpha-defensin in synovial fluid samples. The error bars represent the standard deviation of three independent experiments.

A total of 10 synovial fluid samples sequentially submitted for alpha-defensin and CRP tests by ELISA and our msLFIA devices. The msLFIA cut-off values for alpha-defensin and CRP have been set at 10 μg/mL and 5 μg/mL, respectively. The assay results are shown in Fig. 3(a), alpha-defensin test were positive in 8 cases and were negative in 2 cases, while CRP test were positive in 7 cases and were negative in 3 cases. The ELISA results for detection of alpha-defensin and CRP in synovial fluid samples are shown in Table 1. Eight ELISA-alpha-defensin and seven ELISA-CRP samples with higher value over 21.5 μg/mL and 8.3 μg/mL, respectively, were positive for the msLFIAs, and 2 ELISA-alpha-defensin and 3 ELISA-CRP samples with value below 7.2 μg/mL and 5.4 μg/mL were negative with the LFIAs. Moreover, the optical intensity of alpha-defensin and CRP of the msLFIA test strips analyzed by ImageJ were shown in Fig. 3(b). The results were correlated with the concentration of each synovial fluid sample by ELISA. The correlation coefficient (R^2^) was calculated and presented in Fig. 4. The correlation coefficients of alpha-defensin and CRP between msLFIA and ELISA were 0.91 and 0.92, respectively, which indicate an acceptable agreement between the two detection methods for the protein levels of alpha-defensin and CRP. The signal intensity was dependent on the affinity of antibody to antigen. The slope of the correlation between msLFIA and ELISA was 70.15 and 178.67 for alpha-defensin and CRP, respectively, which indicating that the higher slope of CRP has higher signal intensity than alpha-defensin because anti-CRP antibody possesses a higher affinity for CRP. These results suggested that the msLFIA strip is a reliable test for the detection of alpha-defensin and CRP in synovial fluid samples. It has the advantages of rapid, low-cost, ease of use, and can be used as the preliminary test for evaluating patients with suspected PJI.

**Table 1:** The protein levels of alpha-defensin and CRP in 10 synovial fluid samples analyzed by ELISA.

Sample	Alpha-defensin (µg/mL)	CRP (µg/mL)
001	29.8 ± 0.3	17.3 ± 2.6
002	28.6 ± 0.4	21.7 ± 1.3
003	30.1 ± 0.5	4.0 ± 0.4
004	29.0 ± 0.6	5.4 ± 0.3
005	27.8 ± 1.0	8.3 ± 0.5
006	21.5 ± 0.8	15.7 ± 0.5
007	23.3 ± 1.3	4.9 ± 0.4
008	23.6 ± 0.2	10.2 ± 0.8
009	1.1 ± 0.1	12.6 ± 1.3
010	7.2 ± 1.1	18.6 ± 2.8

**Figure 5:**
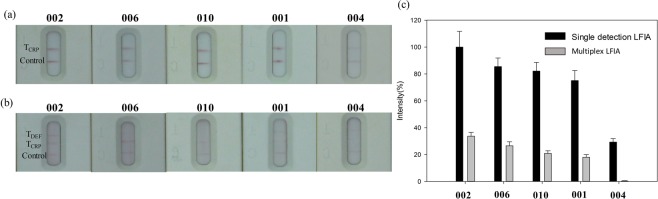
Photographs of test strips showing five synovial fluid samples by (**a**) the conventional single CRP detection LFIA and (**b**) alpha-defensin/CRP msLFIA. (**c**) The normalized optical intensity of synovial fluid samples analyzed using ImageJ. The error bars represent the standard deviation of three independent experiments.

Furthermore, msLFIA device is the multiple analyte detection version of the traditional single-plex LFIA. Trying to accommodate as many test lines as possible within a limited space on a device is a great challenge. Here, we compared the msLFIA with single CRP detection LFIA, the results of test strips by msLFIA (Fig. 5(a)) and traditional single-plex LFIA (Fig. 5(b)) for five synovial fluid samples showed the CRP level from 5.4 to 21.7 μg/mL, and the optical intensity of CRP assay was correlated with the concentration of each synovial fluid sample, which were analyzed by ImageJ (Fig. 5(c)). As expected, the optical intensities of the traditional single-plex LFIA were higher than those of our msLFIA device by 3.0- to 4.2-fold, regardless of the concentration of alpha-defensin is high (29.8 μg/mL, 4.2-fold) or low (7.2 μg/mL, 3.9-fold) in the synovial fluid sample.

## Discussion

The diagnosis of PJI is a most critical step in the management of a patient with a painful arthroplasty by guiding surgical decision-making. However, the currently used laboratory tests for infections are not able to provide a sufficiently accurate and rapid diagnosis of PJI. To address the issue of accuracy and efficiency of PJI diagnosis, multiplex testing is becoming indispensable in contemporary clinical diagnosis. With an increasing numbers of biomarkers being discovered over the years, there is often a need to detect several biomarkers simultaneously to generate meaningful or conclusive information. Furthermore, synovial fluid biomarkers have demonstrated great promise in providing a highly accurate diagnosis of PJI^13–15,17^.

In order to obtain rapid and on-site test results, paper-based diagnostic devices are the most popular POCT tools due to its flexibility, sample fabrication and visualization, cost effectiveness and portability^41–44^. Among them, traditional LFIA biosensor is widely adopted based on its single detection comprised of a test with a control and a test lines as the result output. Recently, multiplex LFIA are proposed for its multi-target detection; for example, toxins^38^ and drugs of abuse^45^. While recognizing the complex nature of human diseases, overlapping symptoms, and states of co-infections, the number of biomarkers linked to certain diseases is often increasing, with multiple biomarkers being assayed to confirm diagnosis. However, analytical sensitivity and specificity of multiplexing may be affected by several issues, for example, the interference between multiple biomarkers and antibodies, different binding kinetics among the antibodies, and buffer system compatibility in the multi-parameter lateral flow detection^46^. In the present study, a msLFIA platform was successful constructed and validated. In addition, a quick preincubation was performed to extend the interaction time between antigens and antibodies, which ensured a complete reaction between the AuNP-antibodies conjugates and the free analytes in the synovial fluid sample, so the samples could be analyzed within 20 min even with small sample volume and not requiring any additional pretreatment steps. This preincubation step increased the sensitivity, accuracy, and reproducibility of the test strip, which could simultaneously qualify alpha-defensin and CRP in synovial fluid.

Compared to using ELISA for detecting alpha-defensin and CRP levels, msLFIA has the potential for screening for these two biomarkers due to its superior efficiency. The advantages of the msLFIA over the ELISA include rapid results, less sample processing requirement, low-cost, yes-no answers generated without additional instruments, and ease of use for non-laboratory personnel. Despite the inability to accurately quantify the analyte, the multiplex platform offers multiple advantages for simplicity, rapidity, sensitivity, cost-effectiveness, and time-efficiency. Herein we described the development and initial validation of the first point-of-care alpha-defensin/CRP multiplex detection device. In our study, six synovial fluid samples (001, 002, and 005–008) were PJI positive and four (003, 004, 009 and 010) were PJI negative, which is summarized in Table 2. The synovial fluid alpha-defensin results alone correctly identified eight patients, corresponding to a specificity of 50% and a sensitivity of 100% for our msLFIA. However, there were two false-positive results for PJI negative. The synovial fluid CRP results of msLFIA were used as a complementary biomarker in order to improve the specificity of alpha-defensin assay alone. When the combined algorithm of synovial fluid alpha-defensin and CRP assays was applied, two false-positive alpha-defensin results (003 and 004) were corrected to true-negative results. Unfortunately, one true-positive sample (007) was turned to false-negative, so the specificity and sensitivity of the combined algorithm were 100% (4/4) and 83.3% (5/6), respectively. In conclusion, using the combined algorithm with our msLFIA, we can correctly diagnosed 90% of the cases as PJI, where the specificity and accuracy were improved from 50% (2/4) to 100% (4/4) and 80% (8/10) to 90% (9/10), respectively, but the sensitivity was decreased from 100% (6/6) to 83% (5/6) due to one false-negative result. In conclusion, this device can detect resuspended alpha-defensin and CRP in synovial fluid samples with the advantages of rapidity, portability, low-cost and easy to use; in addition, it is a feasible diagnostic test for PJI, and it has the potential to influence decision making without additional expensive diagnostic workup.

**Table 2:** Summary of the PJI diagnosis, the alpha-defensin and CRP results of msLFIA for 10 synovial fluid samples.

Sample	Results of msLFIA		PJI diagnosis
	Alpha-defensin	CRP	
001	+	+	+
002	+	+	+
003	+	−	−
004	+	−	−
005	+	+	+
006	+	+	+
007	+	−	+
008	+	+	+
009	−	+	−
010	−	+	−

## Methods

### Reagents and equipment

Methanol (99%), 2-propanol (99%), ethanol (99%), trisodium citrate (>99%), hydrogen tetrachloroaurate (III) trihydrate (99%), borate buffer, sucrose, and CRP were purchased from Sigma-Aldrich (St. Louis, MO, USA). Anti-CRP antibody was purchased from Arista Biologicals Inc. (Allentown, PA, USA). Anti-alpha-defensin antibody, Human alpha-defensin 1 DuoSet ELISA and Human C-Reactive Protein/CRP DuoSet ELISA were obtained from R&D systems (Minneapolis, MN, USA), Goat anti-mouse immunoglobulin (IgG) antibody was supplied by Jackson ImmunoResearch Laboratories (West Grove, PA, USA). We used ultrapure water (18.2 mΩ·cm) throughout the experiments, which was filtered through a Milli-Q system (Millipore, Milford, MA, USA). Nitrocellulose membrane (NC membrane) was purchased from Sartorius Stedim Biotech GmbH (Goettingen, Germany). The sample pad, conjugate pad, absorbent pad, adhesive backing card, and stacking pads were purchased from ShangHai GoldBio Co. Ltd. (Shanghai, China). The T_DEF_-, T_CRP_ and C-lines on the NC membrane were prepared on the membranes using a Lateral Flow Reagent Dispenser (Claremont BioSolutions, Upland, CA) to dispense the anti-alpha-defensin, anti-CRP antibody and goat anti-mouse IgG antibody, respectively. The assembled assay was cut into individual strips of 4 mm in width using a Rapid Test Cutter ZQ2000 (Shanghai kinbio Tech Co., Ltd, China), enabling the platform to be loaded in a commercially available plastic cassette.

### Preparation of AuNPs

AuNPs were synthesized using the Turkevich method^47^. Briefly, 30 nm diameter AuNPs were prepared as follows: A 250 mL two-neck round bottom flask was filled with 100 mL of ultrapure water and placed on a magnetic stirring heater. After boiling, 1 mL of 1% (w/v) trisodium citrate was added with vigorous stirring. After 10 min, 1.2 mL of 0.1% (w/v) chloroauric acid was added to the solution, which changed the color of the solution from yellow to black and then red. We continued to heat and stir the solution for another 10 min and then cooled the sample in a water-ice bath for 30 min.

### AuNP-anti-alpha-defensin and anti-CRP antibody conjugate preparation

The AuNP-anti- alpha-defensin and anti-CRP conjugates were prepared with the same procedure for AuNPS. 1 mL AuNPs was mixed with 5 μg antibody in 0.01 M amine-free PBS solution at pH 8.4. The anti- alpha-defensin or CRP antibodies were attached to the surface of the AuNPs over the 90 min incubation period via multiple interactions. Then 0.1 M tris-buffered saline (TBS) with 0.1% (w/v) Tween 20 was added to terminate the antibody/AuNP binding reaction. To remove any excess antibodies, 0.01 M TBS with 0.1% (w/v) Tween 20 was added at 5-times the volume of the conjugate mixture and centrifuged at 8000 *g* for 35 min. The final conjugates were reconstituted in 0.01 M TBS containing 2% (w/v) bovine serum albumin (BSA), 10% sucrose and 0.1% NaN_3_ after removal of the supernatant. The conjugates were stored at 4 °C until use. To prepare the conjugate pads for the alpha-defensin and CRP assay, the conjugates were first diluted to 0.060 O.D. in the conjugate buffer (2 mM borate buffer with 5% sucrose).

### Fabrication of the msLFIA test strips and test procedure

The LFIA strips consisted of five parts as follows: sample pad, conjugate pad, stacking pad, NC membrane, and absorbent pad (Fig. 2(a)). For preparing the test zones, the anti-alpha-defensin, and anti-CRP and goat anti-mouse immunoglobulin (1.5, 0.6, and 1 mg/mL, respectively) were separately dispensed onto the NC membrane in turn at a rate of 0.7 μL/cm to generate two test lines and one control line. The three lines were positioned at a 2.5 mm interval. Finally, the sample pad, conjugate pad, stacking pad, NC membrane, and absorbent pad were laminated onto a plastic backing and divided into strips.

### Validation with clinical synovial fluid sample

All clinical samples were obtained with written informed consent, and the study design was approved by the Institutional Review Board of Chang Gung Medical Foundation, which is organized and operates in accordance with Good Clinical Practice and the applicable laws and regulations (IRB No. 201701976B0). All the test processes were performed in accordance with the relevant guidelines and regulations. A total of 10 synovial fluid samples were tested for clinical validation of the msLFIA. The synovial fluid samples were collected from patients who underwent total hip or knee replacement due to PJI or implant loosening. A quick preincubation^40^ was performed for preparing the conjugate pad. Briefly, two AuNPs labeled antibodies were pre-mixed together with an equal volume of 5 μL. Afterwards, 1 μL of the synovial fluid sample was mixed with 10 μL of the AuNPs conjugate mixture and then pipetted onto the conjugate pad. For running a single assay, a mixture of 1 μL of the synovial fluid sample and 5 μL of the AuNPs-anti-CRP conjugate was used. The LFIA strip was installed to be loaded into a commercially available plastic cassette and then dipped into 100 μL of running buffer. After loading each sample and waiting for 20 min, the color intensities at the T- and C-lines of the strips were captured using a CCD camera and analysed using ImageJ software^48^ (National Institutes of Health, Bethesda, MD). The intensity was calculated by measuring the ROI areas of 2 × 2 mm^2^ at the center of T_DEF_- and T_CRP_-line of the msLFIA strip and then subtracting the mean background ROI of two adjacent areas at the distance of 0.5 mm from T-line in opposite direction. For comparative purposes, the alpha-defensin and CRP protein levels in synovial fluid samples from each patient was collected in the standard laboratory method. Levels of alpha-defensin and CRP in the synovial fluid were measured using Human alpha-defensin 1 DuoSet ELISA and Human C-Reactive Protein/CRP DuoSet ELISA, respectively, according to the manufacturer’s instructions.
